# Feasibility of Self-Directed Learning of Cardiopulmonary Resuscitation Skills Using Interactive Video in SimZone 0: Pilot Randomized Educational Trial

**DOI:** 10.2196/95529

**Published:** 2026-08-27

**Authors:** Álvaro Trampal Ramos, Guillermo Charneco Salguero, Belén González-Tejerina, Elisa Moreno-Palacios, César Leal-Costa

**Affiliations:** 1 Department of Nursing, Universidad San Pablo-CEU, CEU Universities, Urbanización Montepríncipe, Boadilla del Monte, Madrid, 28668, Spain; 2 Fundación Jiménez Díaz School of Nursing, Universidad Autónoma de Madrid, Madrid, Spain; 3 Breast Pathology Unit, Hospital Universitario La Paz, Madrid, Spain; 4 Department of Nursing, Universidad de Murcia, Murcia, Spain

**Keywords:** self-directed learning, SimZones, simulation, interactive video, cardiopulmonary resuscitation, CPR, competence, nursing, nursing students, nursing education

## Abstract

**Background:**

The SimZones model is an organizational framework for simulation-based education that structures learning across 5 progressive zones, from self-directed preparatory activities (zone 0) to team-based clinical scenarios (zones 1‐4). However, empirical evidence on the impact of zone 0 regarding procedural skill acquisition and retention remains limited.

**Objective:**

The present pilot study aimed to build on this evidence gap by examining whether the addition of a structured zone 0 self-directed preparatory phase, delivered through interactive video (IV), could enhance cardiopulmonary resuscitation (CPR) competence acquisition, skill retention, and CPR quality among nursing students when combined with conventional instructor-led zone 1 training, while also assessing the feasibility and acceptability of the intervention.

**Methods:**

A total of 52 nursing students from San Pablo CEU University (Madrid, Spain) were randomly assigned to an experimental group (n=33, SimZone 0 IV followed by SimZone 1 instructor-led seminar) or a control group (n=19, SimZone 1 instructor-led seminar only). CPR competence acquisition, skill retention, CPR quality, and feasibility were assessed immediately after zone 1 training and at 3 and 6 months.

**Results:**

In the self-directed zone 0 phase, 73% (24/33) of experimental group students achieved competence. After zone 1 training, 100% (28/28) of the experimental group achieved competence vs 84% (16/19) of the control group (*P*=.06, Fisher exact test). At 3 months, competence was 96% (23/24) vs 71% (10/14; *P*=.05), and at 6 months, 96% (22/23) vs 62% (8/13; *P*=.02). Mean CPR quality scores were consistently higher in the experimental group across all time points (zone 1: 94%, SD 11.8% vs 90%, SD 16.4%; 3 months: 92%, SD 10.9% vs 90%, SD 12.1%; and 6 months: 92%, SD 10.9% vs 85%, SD 17.8%). The intervention was feasible, well accepted, and free of adverse events or technical issues.

**Conclusions:**

Self-directed IV learning as a preparatory phase before instructor-led training is feasible, acceptable, and is associated with preliminary signals of enhanced CPR skill acquisition and retention among nursing students. These findings should be interpreted with caution given the pilot nature of the study and the small sample size.

## Introduction

Clinical simulation is a methodology that allows people to experience a representation of a real health care event in order to practice, learn, evaluate, test, or understand systems or human actions [1]. Beyond technical competence, clinical simulation promotes critical thinking, decision-making, and teamwork skills, while offering a safe space to make mistakes and learn from them, a feature that is particularly crucial in the training of future health care professionals [2]. Within this context, the SimZones framework proposed by Roussin and Weinstock [3,4] provides a structured approach that organizes simulation-based learning across 5 progressive zones. Zone 0 focuses on self-directed learning with automated feedback tools such as interactive video (IV); zone 1 delivers instructor-led basic skill training; zone 2 engages learners in acute simulation scenarios; zone 3 is aimed at team and systems development; and zone 4 is intended for briefings associated with actual patient care.

IV is particularly well suited to zone 0 because it allows learners to control their own learning pace, make decisions at critical moments, and receive immediate automated feedback [5-7]. In many institutions, however, simulation programs begin directly in zone 1 without prior zone 0 exposure, missing the opportunity for structured preparatory self-directed learning [8,9]. Incorporating zone 0 into simulation programs, therefore, represents a key pedagogical opportunity to foster prior learner preparation through deliberate repetition and familiarization with procedures.

Cardiopulmonary resuscitation (CPR) is a critical clinical skill that all health care professionals must master, as its timely and correct application is essential in cardiac arrest [10]. To support CPR skill development in zone 1, several instructor-led feedback approaches have been used, including the pause and reflect technique [11], deliberate rapid-cycle practice [12], and the 4-step methodology developed by Peyton [13,14]. The approach of Peyton (comprising demonstration, deconstruction, comprehension, and execution) has shown effectiveness in teaching complex procedural skills by breaking them into manageable steps. When adapted to an IV format for zone 0, this methodology provides a structured framework for self-directed CPR learning before transitioning to instructor-led zone 1 practice.

In recent years, health sciences training has evolved toward more student-centered models where learners take a leading role in acquiring competencies [15,16], aligning with the need to promote active and autonomous learning in critical clinical skills such as CPR [17]. This approach is grounded in constructivist learning theory, which emphasizes active knowledge construction through guided practice [18], and is complemented by Bandura [19] self-efficacy theory, which highlights the role of perceived competence as a motivational driver in autonomous learning contexts. Together, these frameworks suggest that a structured zone 0 IV preparatory phase may enhance both acquisition and retention of CPR competencies when combined with conventional zone 1 training.

Although some innovative approaches have explored self-directed virtual learning experiences [20], empirical evidence specifically assessing the effectiveness of zone 0 in developing clinical competencies remains limited. No studies have directly evaluated the added value of zone 0 IV-based preparatory learning on CPR competence acquisition and retention in undergraduate nursing students. Building on this evidence gap, the present pilot study aimed to examine whether adding a structured zone 0 self-directed preparatory phase, delivered via IV, could enhance CPR competence acquisition, skill retention, and CPR quality when combined with conventional instructor-led zone 1 training, while also assessing the feasibility and acceptability of the intervention. The hypothesis is that zone 0 IV training would result in a higher proportion of students acquiring and maintaining CPR competencies compared with zone 1 training alone.

## Methods

### Study Design

A pilot randomized educational trial was conducted to evaluate the feasibility and preliminary effectiveness of adding a self-directed SimZone 0 preparatory phase using IV prior to instructor-led CPR training in SimZone 1. The study was conducted between December 2023 and September 2024. The educational intervention was structured according to the 4-step approach developed by Peyton [13,14], adapted to a self-directed learning format for the SimZone 0 phase and to instructor-led training for SimZone 1. The primary outcome was CPR competence acquisition, assessed immediately after SimZone 1 training, at 3 months, and at 6 months. The secondary outcome was the CPR quality score, assessed at the same time points. Feasibility and acceptability were assessed as complementary pilot objectives, including recruitment rate, participation rate, intervention adherence, absence of adverse technical events, student satisfaction, and perceived learning.

The trial followed the CONSORT (Consolidated Standards of Reporting Trials) extension for pilot and feasibility studies [21]. The completed checklist is provided as Checklist 1.

Although registration is not a legal or institutional requirement for educational research in Spain, the study was retrospectively registered as a pilot randomized educational trial in the ISRCTN registry (identifier: ISRCTN16191678) on June 26, 2025 [22]. This registration was performed voluntarily to promote transparency and adherence to international reporting standards. The study design, methodology, and analytical plan were established prior to participant recruitment and were not modified during the course of the study. The registered record accurately reflects the study as conducted; no protocol amendments were introduced.

### Sample Size Calculation

As this was a pilot study, the sample size was intended to provide preliminary estimates of feasibility and effect size rather than to support definitive hypothesis testing.

An a priori power calculation was performed using G*Power 3.1 (Heinrich Heine University Düsseldorf), assuming a moderate effect size (*f*=0.40) according to Cohen conventions [23], a 2-group comparison, and a 95% CI. This resulted in an estimated minimum total sample size of approximately 50 participants. To account for potential attrition during follow-up, particularly in the experimental group, additional participants were recruited for that group. This approach is consistent with methodological recommendations for pilot and feasibility trials, in which unequal group sizes may be acceptable to ensure sufficient data for preliminary analyses.

### Participants

Participants were recruited during the 2023-2024 academic year through direct in-class announcements at San Pablo CEU University (Madrid, Spain), with no academic or financial incentive offered for enrollment. To be eligible, participants had to be enrolled in the first or second year of the degree in nursing and not have previously received formal training in CPR. Students were excluded if they did not voluntarily agree to participate, if they presented migraines, motion sickness, vestibular alterations, or other conditions that prevented participation on the day of practice, or if they had significant difficulties understanding the proposed activity.

Before the intervention, a brief ad hoc questionnaire was administered to verify the absence of previous CPR training. This screening tool was not a validated psychometric instrument but a pragmatic 4-item questionnaire asking whether the participant had completed any formal or informal CPR course, the name of the institution providing the course, the year of completion, and the approximate duration. No additional items were included beyond these 4. Participants who reported having received prior CPR training were excluded from the study. A total of 52 nursing students voluntarily enrolled in the study; no participants were excluded on the basis of prior CPR training, as all confirmed having no prior experience before enrollment.

### Randomization and Blinding

Participants were recruited on a voluntary basis and subsequently randomized into either the experimental or the control group. Allocation was performed using simple randomization generated with GraphPad QuickCalcs (Dotmatics, [24]) by an external researcher who was not involved in training or assessment.

Given that the experimental group was required to complete an additional self-directed learning session in zone 0 prior to zone 1 training, a higher attrition rate was anticipated for this group. To ensure sufficient data for preliminary analyses at all assessment time points, an unequal allocation ratio of approximately 2:1 (experimental:control) was prespecified, so that more participants would be randomized to the experimental group (33 experimental vs 19 control). This approach is consistent with methodological recommendations for pilot and feasibility trials in which unequal allocation may be justified to compensate for anticipated differential attrition.

Blinding was applied during the assessment stage. Instructors responsible for evaluating CPR performance were unaware of participants’ group allocation, which remained concealed until the completion of data analysis.

### Training Program

Table 1 presents the components received by each group, including the timing, duration, and content of each educational phase.

**Table 1.** Components of the educational intervention by group.

Component	Timing	Duration	Content	Control	Experimental
Zone 0: IV^a^	Before zone 1	∼30 minutes	Self-directed IV using the 4 steps developed by Peyton, automated feedback, and hands-on practice with Laerdal QCPR^b^	No	Yes
Zone 1: instructor-led seminar	Main session	4 hours	ERC^c^, BLS^d^ protocol, instructor demonstration, guided practice, and real-time QCPR feedback	Yes	Yes
Competence assessment	After zone 0, 1, 3, and 6 months	∼10 minutes	Modified ERC BLS checklist+CPR^e^ quality metrics	Yes	Yes

a IV: interactive video.

b QCPR: QualityCPR (Laerdal Medical automated feedback system).

c ERC: European Resuscitation Council.

d BLS: Basic Life Support.

e CPR: cardiopulmonary resuscitation.

#### Experimental Group Intervention

**Figure 1.**
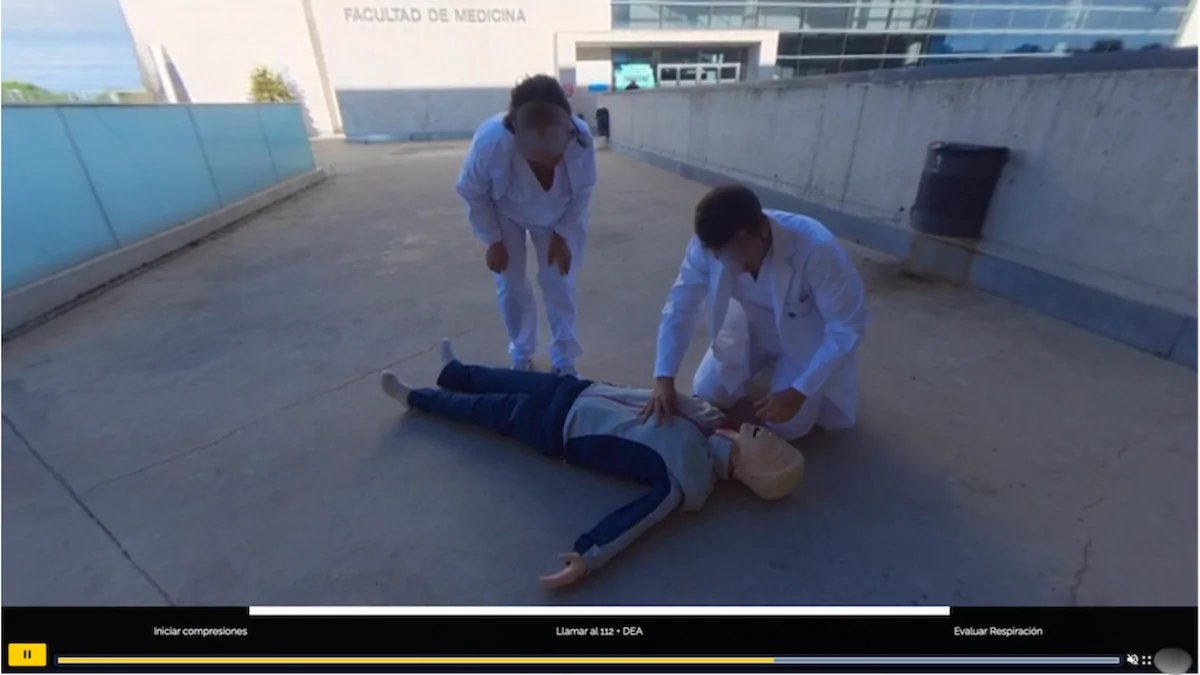
Screenshot of an interactive video created with the Stornaway.io application.

Students in the experimental group completed an individual CPR training session in zone 0, lasting approximately 30 minutes, before attending the zone 1 seminar (Table 1). The zone 0 session followed a learning sequence based on the 4-step methodology developed by Peyton, delivered through an IV developed using the Stornaway.io platform:

1. Uninterrupted demonstration: students first watched a complete video demonstrating the basic CPR sequence without interruptions.
2. Step-by-step explanation: they then viewed the same video with pauses at key points in the CPR procedure, with explanations of each technique and its rationale.
3. Guided decision-making: students watched the video again and were prompted to make decisions at each critical point (Figure 1). Two or three options were presented at each decision point. If an incorrect option was chosen, the video provided immediate feedback and explained the correct response. If the correct option was selected, the video continued to the next scene. Students were allowed to repeat the video as many times as desired.
4. Hands-on practice with feedback: after completing the IV, students practiced the complete CPR sequence using the Little Anne (Laerdal Medical) task trainer equipped with the QCPR (Quality CPR) system, which provided real-time automated feedback on chest compressions prior to the final assessment.
5. Competency assessment: once the zone 0 training sequence was completed, students underwent a CPR competency assessment supervised by an instructor. No feedback was given before or after the evaluation.

**Figure 2.**
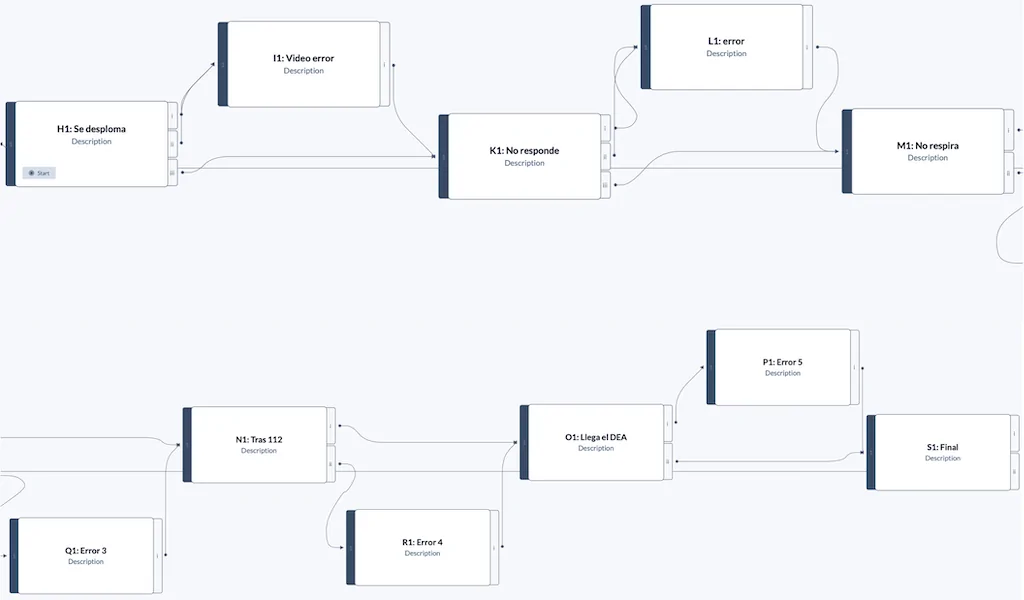
Screenshot of the cardiopulmonary resuscitation (CPR) scenario diagram generated in Stornaway.io.

The interactive resource was developed using Stornaway.io, a digital platform that allows the creation of branched narrative video structures, an interactive format in which users make decisions that modify the course of the content [25]. In this case, the video guides the learner through a simulated CPR scenario, requiring them to choose among several on-screen options. When a correct action is selected, the video continues to the next step in the sequence. If the student selects an incorrect option, the video redirects to a feedback segment, which explains the error and provides guidance on the correct response. The decision points embedded in the video align with the critical moments defined by the European Resuscitation Council (ERC) for CPR skills assessment: verification of patient response, assessment of breathing, activation of emergency services using the corresponding telephone number, initiation of effective chest compressions, and application of the automated external defibrillator (Figure 2). These decisions reflect the criteria that would later be used in the practical evaluation of the students.

Students accessed the video from the simulation room on a computer with internet access. An instructor was present to assist them with connecting to the Stornaway.io platform and providing technical support if needed.

#### Control Group Intervention

Students assigned to the control group completed only the zone 1 instructor-led CPR seminar, with no prior zone 0 preparatory phase. The seminar lasted 4 hours and was delivered at a ratio of 1 instructor to 9 students, each with access to a CPR torso. The seminar followed the standards of the ERC [15], and the instructor used the 4-step methodology developed by Peyton to teach the CPR sequence. All seminars were delivered by the same ERC/American Heart Association (AHA)-certified instructor to ensure consistency and minimize instructor bias.

Both groups received hands-on CPR training in zone 1 with real-time feedback during practice, including verbal guidance from the instructor and automated performance data from the simulators’ integrated feedback systems. No feedback was provided before or after the formal competency evaluation. The total instructional time, therefore, differed between groups: the experimental group received approximately 30 additional minutes of zone 0 self-directed learning in addition to the 4-hour zone 1 seminar received by both groups.

### Evaluation Instrument

For the assessment of CPR competence, we used a modified version of the ERC Basic Life Support course assessment checklist [26], which is based on the key points outlined in the 2021 ERC guidelines for basic life support [27].

This evaluation was performed after the end of the self-study session of the experimental group in zone 0 and after the end of the CPR session in zone 1 with the experimental group and the control group. This competency assessment was repeated at 3 and 6 months.

In order to be considered competent in CPR, students were required to correctly complete all 11 points on the evaluation checklist, reflecting the mastery-based nature of Basic Life Support certification standards. Each item was scored as “correct” or “incorrect” (Textbox 1).

Textbox 1. **Cardiopulmonary resuscitation (CPR) competency checklist adapted for the European Resuscitation Council and modified by the author.**

**Competence**

- Verify response
- Assesses breathing (demonstrates head tilt and chin lift)
- Assesses breathing (demonstrates looking, listening, and feeling for normal breathing for no more than 10 s)
- Call emergency services
- Chest compressions
- Activate automated external defibrillator
- Apply chest patches
- Do not touch the patient. Allow rhythm analysis while making sure no one touches the victim
- Shock
- Follow automated external defibrillator instructions
- CPR

In this study, CPR quality was evaluated using integrated sensors in the Laerdal Little Anne task trainer equipped with the QCPR system [28]. This system evaluates several key performance indicators: proper chest re-expansion after compressions, compression depth within the recommended range of 5 cm to 6 cm, compression rate between 100 and 120 compressions per minute, duration of interruptions, and the chest compression fraction, defined as the percentage of time during CPR in which chest compressions are actively performed [29]. These metrics are automatically recorded and used to generate an overall performance score in percentage terms. A quality score of 70% or higher was considered acceptable CPR performance, in line with ERC recommendations [27]. Several studies have used the Little Anne QCPR (Laerdal Medical) manikin as a CPR training tool. For example, Smart et al [30] evaluated the impact of real-time feedback and competition between trainees during training sessions with QCPR-equipped manikins, observing that the use of these devices significantly improves the quality of chest compressions performed by participants. Likewise, Dine et al [31] demonstrated that the combination of feedback provided by CPR training devices and subsequent debriefing contributes to improving the overall quality of CPR. In addition, the use of the Laerdal Little Anne QCPR system is aligned with official ERC and AHA recommendations for the measurement of CPR quality indicators, and its reliability and validity in assessing key resuscitation parameters are widely supported by international studies [32]. The scoring system is based on objective sensors that assess parameters such as depth of compressions, rate, recoil, and compression fraction (percentage of time during CPR in which active chest compressions are being performed on the patient, relative to the total time of cardiac arrest), following the manufacturer’s guidelines described in the supporting documentation of Laerdal, which align with evidence-based resuscitation standards [28].

Student satisfaction and perceived learning were assessed using an ad hoc Likert scale questionnaire (1‐5, where 5=“maximum satisfaction”) administered at the end of the zone 0 session and after the zone 1 seminar. The questionnaire evaluated overall satisfaction with the activity, satisfaction with the methodology, perceived learning acquisition, self-confidence to perform CPR, and willingness to recommend the approach to others. At 3 and 6 months, participants additionally rated their confidence in skill retention on the same scale.

### Ethical Considerations

#### Ethics Committee Approval

This study was approved by the Ethics Committee of CEU San Pablo University, Madrid, Spain (approval number: 768/23/89). All study procedures were conducted in accordance with the ethical principles outlined in the Declaration of Helsinki.

#### Informed Consent

All participants provided written informed consent prior to enrollment. Participants were informed that their participation was voluntary and that they could withdraw at any time without academic or personal repercussions. Potential risks identified were possible mild psychological stress associated with performance evaluation and fatigue during CPR practice. To mitigate these risks, training sessions were conducted in a safe and supportive environment, and breaks were provided when necessary.

#### Privacy and Confidentiality

Data were collected using anonymized digital forms (Microsoft Forms), with each participant identified only by a randomly assigned code. No personally identifiable information was associated with the responses. All data were stored securely in the institutional Microsoft 365 account with access restricted to the principal investigator and were analyzed in aggregate form.

#### Participant Compensation

No financial or academic compensation was offered or provided to participants for their collaboration in the study.

### Data Analysis

Statistical analysis was performed using SPSS software (version 29, IBM). Descriptive statistics were calculated for competence outcomes (the proportion of participants achieving full checklist competence) and for CPR quality scores at each assessment time point: after zone 1 training, at 3 months, and at 6 months. Categorical variables were described using absolute frequencies and percentages.

Normality was assessed using the Shapiro-Wilk test for each continuous variable. Results indicated nonnormal distribution across groups and time points: in the experimental group, Shapiro-Wilk values ranged from *W*=0.634 to *W*=0.651 (all *P*<.001); in the control group, normality could not be computed at zone 1 and at 3 months due to insufficient score variance, and *W*=0.802 (*P*=.007) at 6 months. Given the pilot nature of the study, the small sample size, and the nonnormal distribution of the data, nonparametric analyses were used throughout.

Given that CPR competence acquisition was the primary outcome and was assessed as a binary variable (competent or not competent), between-group comparisons of competence proportions were performed using Fisher exact test at each assessment time point. Odds ratios (OR) with 95% CIs were reported as effect size measures, except at zone 1 where the OR was not estimable due to a zero cell. CPR quality scores, being continuous variables, were compared using the Mann-Whitney *U* test, with the *U* statistic, *z* score, effect size *r* (*r*=Z/√N), and 95% CIs reported. Statistical significance was set at *P*<.05.

Missing data arising from nonattendance at scheduled assessments were handled using a complete case approach. No imputation was performed.

## Results

### Participant Characteristics

Initially, 52 participants were enrolled (19 in the control group and 33 in the experimental group). Baseline characteristics of both groups are presented in Table 2. The groups were broadly comparable in terms of sex distribution (control: 17/19, 89% female participants; experimental: 27/33, 82% female participants) and year of study (control: 7/19, 37% first-year; experimental: 15/33, 45% first-year). A difference in age distribution was observed between groups, with the control group concentrated in the youngest categories and the experimental group showing a wider distribution, as detailed in Table 2. Age was collected using grouped response categories rather than exact continuous values; therefore, formal comparison of mean age between groups was not possible. Given the small cell counts in several categories, this difference was interpreted cautiously and is discussed further in the *Limitations* section.

**Table 2.** Baseline characteristics of study participants.

Variable	Control (n=19), n (%)	Experimental (n=33), n (%)
Age (y)		
18‐20	15 (79)	16 (48)
21‐22	4 (21)	6 (18)
23‐25	0 (0)	5 (15)
26‐28	0 (0)	4 (12)
>40	0 (0)	2 (6)
Sex		
Female	17 (89)	27 (82)
Male	2 (11)	6 (18)
Year of study		
First year	7 (37)	15 (45)
Second year	12 (63)	18 (55)

**Figure 3.**
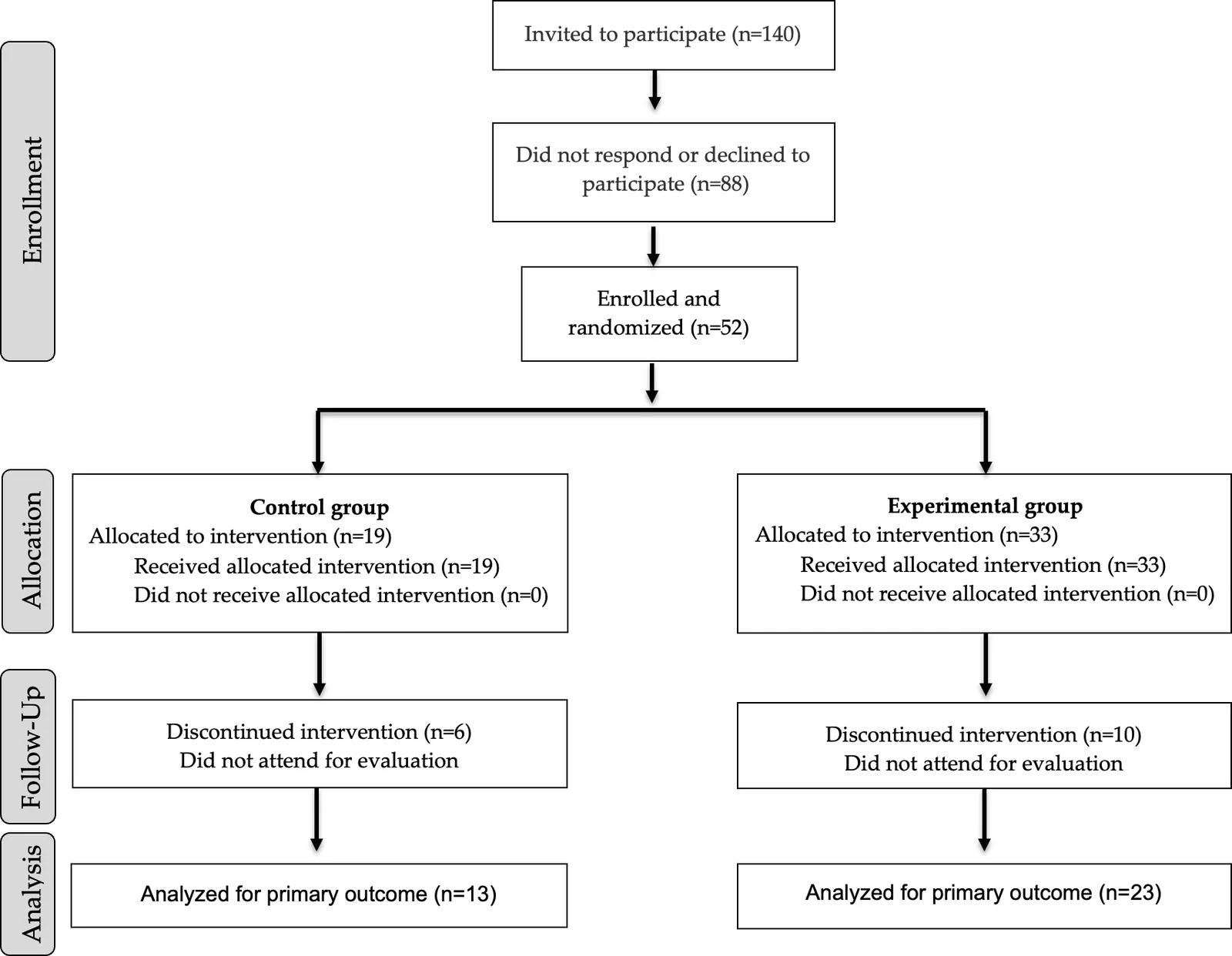
Flow diagram of the study following CONSORT (Consolidated Standards of Reporting Trials) guidelines, illustrating recruitment, randomization, losses due to nonattendance for evaluation, and final analysis.

The number of participants included in each analysis, participant flow, and attrition are shown in the CONSORT flow diagram (Figure 3). A total of 6 participants were lost to follow-up in the control group (5 between zone 1 and 3 mo; 1 between 3 mo and 6 mo) and 10 in the experimental group (5 between zone 0 and zone 1; 4 between zone 1 and 3 mo; 1 between 3 and 6 mo), leaving 13 and 23 participants, respectively, at 6 months. Losses were primarily due to nonattendance at scheduled assessments coinciding with university examination periods.

### Feasibility Outcomes

A total of 52 out of 140 (37%) invited students enrolled in the study. All 33 experimental group participants completed the zone 0 session without technical issues on the Stornaway.io platform or with the Laerdal QCPR equipment. The zone 0 session lasted approximately 30 minutes per participant. No participants withdrew due to adverse events or dissatisfaction with the intervention.

Satisfaction ratings were highly positive in both phases. In zone 0, 100% (33/33) of experimental group participants rated the activity, the methodology, and its applicability to other subjects with the maximum score (5/5). In zone 1, 96% (22/23) of experimental group participants rated the activity with a score of 5, and 4% (1/23) with a score of 4. Regarding methodology, 96% (22/23) awarded the maximum score, and 100% (23/23) recommended its application to other subjects (22/23, 96% with a score of 5 and 1/23, 4% with a score of 4).

Regarding perceived learning after zone 0, 91% (30/33) of students in the experimental group reported having acquired substantial knowledge and skills. Additionally, 85% (28/33) considered that the training significantly improved their competencies, and a similar proportion expressed feeling confident or fairly confident to perform CPR in clinical practice. After zone 1 training, 100% (23/23) of participants reported having acquired CPR knowledge and skills, and 96% (22/23) felt highly or fairly confident to apply the technique.

At 3 months, 70% (26/37) of participants across both groups rated their confidence in skill retention at 4 or 5 out of 5, although 30% (11/37) expressed more moderate confidence levels. At this time point, 49% (18/37) reported having forgotten at least one step of the patient assessment sequence, attributing this primarily to a lack of regular practice. At 6 months, confidence in skill retention improved markedly: 90% (28/31) rated their confidence at 4 or 5, and only 3 participants reported having forgotten any step, suggesting sustained confidence in perceived skill retention over time.

### Competence Acquisition and Retention

The experimental group achieved 73% (24/33) competency acquisition in the self-directed learning phase (zone 0). The control group did not participate in this phase (not applicable).

**Figure 4.**
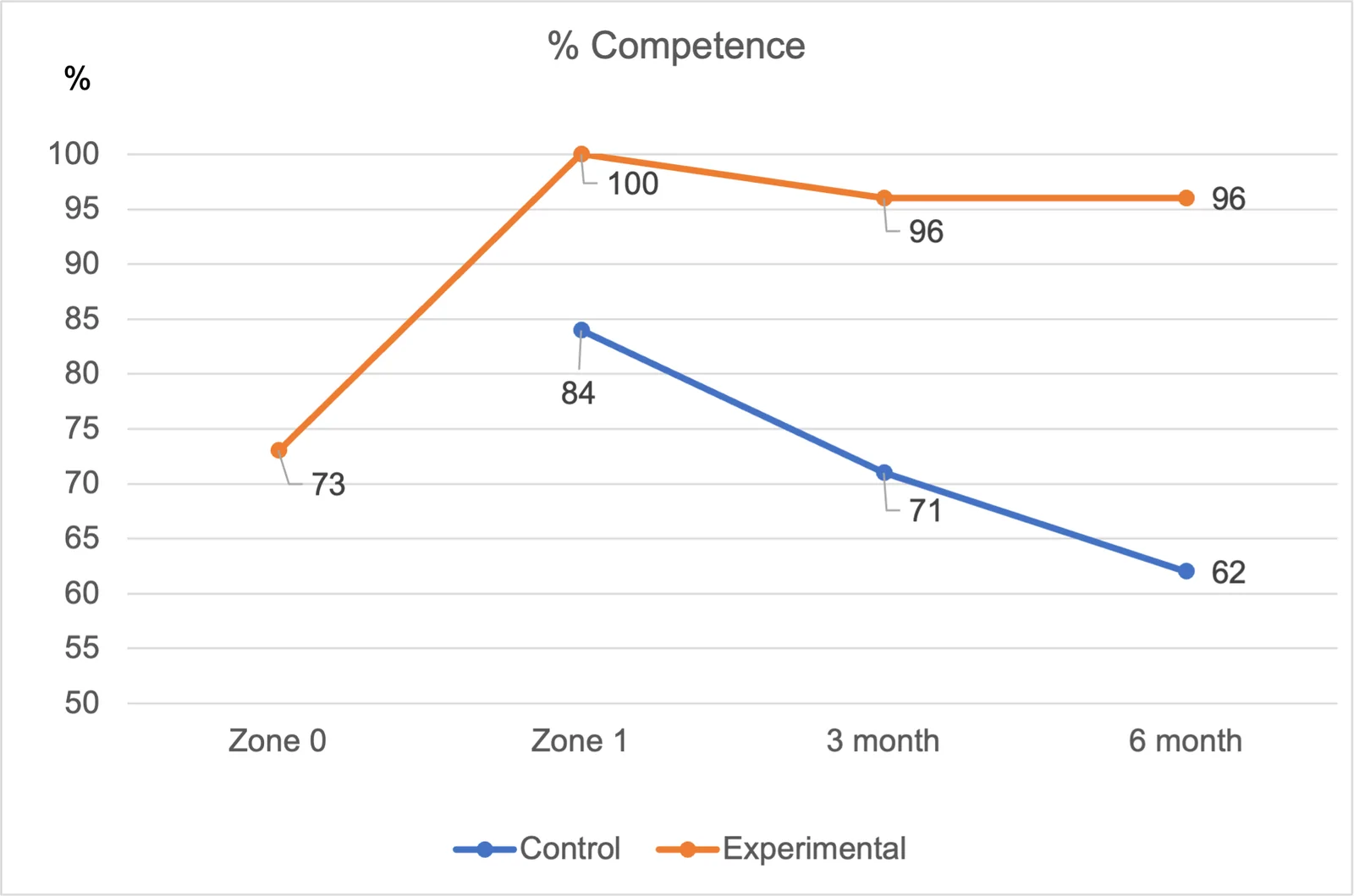
Percentage of competency acquisition in the control and experimental groups in SimZone 0 and SimZone 1, and at the 3-month and 6-month follow-ups.

Between-group comparisons using the Fisher exact test showed that competence differences favoring the experimental group increased over time. Immediately after zone 1 training, the difference did not reach statistical significance (*P*=.06), although all experimental group participants achieved competence (28/28, 100%) compared with 84% (16/19) in the control group. At 3 months, the difference remained marginally nonsignificant (*P*=.05; OR 9.20, 95% CI 0.91‐93.02). At 6 months, the difference was statistically significant (*P*=.02; OR 13.75, 95% CI 1.39‐136.39), with the experimental group showing markedly higher competence retention. Overall retention rates at 6 months were 68% (13/19) in the control group and 70% (23/33) in the experimental group (Table 3, Figure 4).

**Table 3.** Competence levels in the control and experimental groups at each evaluation point^a^.

Time period and group	Participants, n	Competent, n (%)	95% CI	Not competent, n (%)	Fisher *P* value	OR (95% CI)
Zone 0					N/A^b^	N/A
Control	N/A	N/A	N/A	N/A		
Experimental	33	24 (73)	54.5‐86.7	9 (27)		
Zone 1					.06	Not estimable
Control	19	16 (84)	60.4‐96.6	3 (16)		
Experimental	28	28 (100)	87.7‐100	0 (0)		
3 month					.05	9.20 (0.91‐93.02)
Control	14	10 (71)	41.9‐91.6	4 (29)		
Experimental	24	23 (96)	78.9‐99.9	1 (4)		
6 month					.02	13.75 (1.39‐136.39)
Control	13	8 (62)	31.6‐86.1	5 (38)		
Experimental	23	22 (96)	78‐100	1 (4)		

a *P* values are from Fisher exact test (2-sided). The odds ratio (OR) for zone 1 was not estimable due to a zero cell (100% competence in the experimental group). Statistical comparisons were not performed for zone 0 because the control group did not participate in this phase.

b N/A: not applicable.

### CPR Quality Outcomes

**Figure 5.**
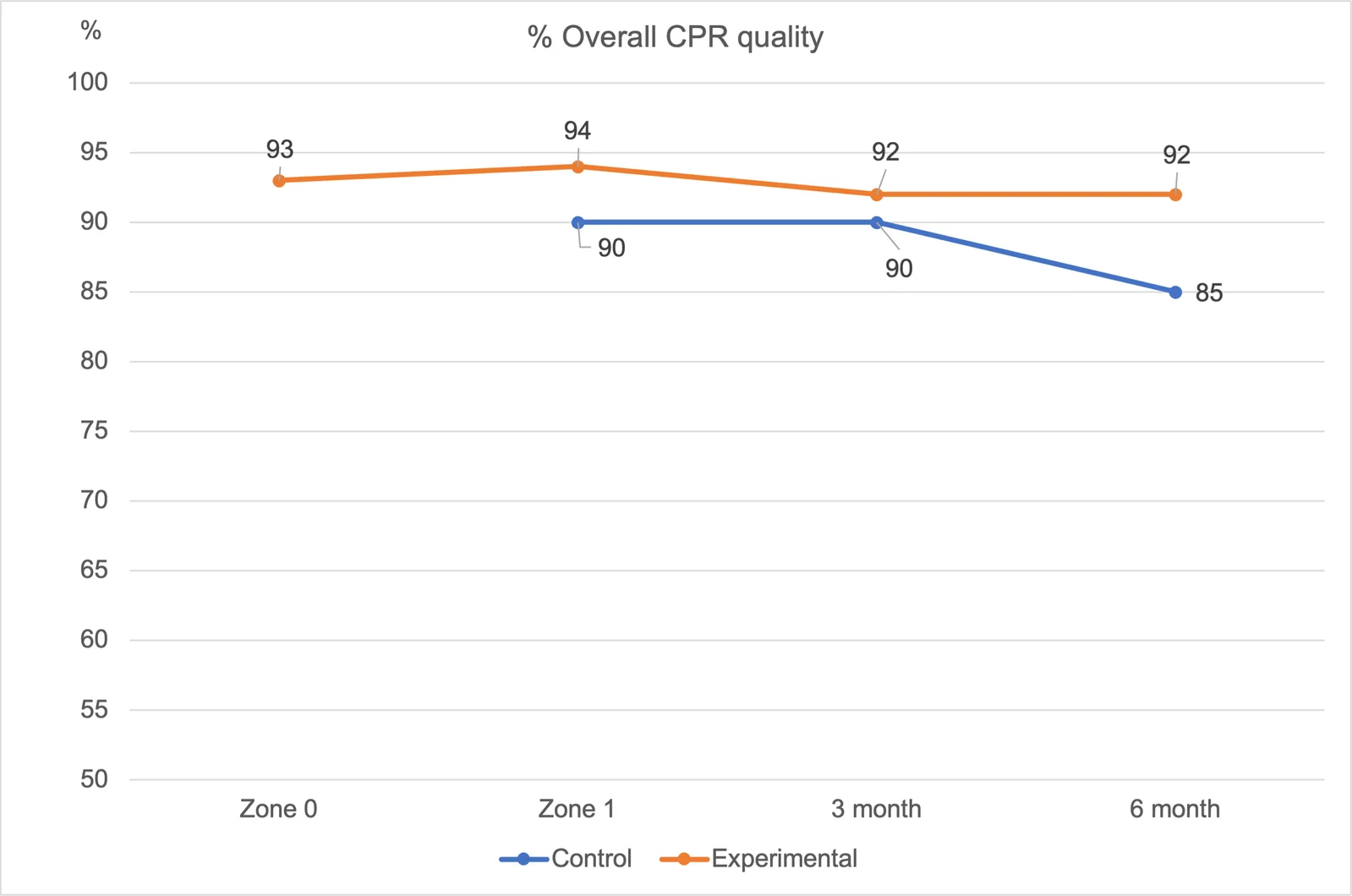
Overall cardiopulmonary resuscitation (CPR) quality scores in the control and experimental groups at each evaluation period.

The experimental group consistently outperformed the control group in total CPR quality scores across all assessment time points, with the most notable differences emerging at the follow-up assessments. Detailed CPR quality metrics are presented in Table 4 and Figure 5.

**Table 4.** Cardiopulmonary resuscitation (CPR) quality metrics in control and experimental groups at each evaluation period.

Metric	Zone 0		Zone 1		3 months		6 months	
	Control	Experimental	Control	Experimental	Control	Experimental	Control	Experimental
Compression release score (%)	N/A^a^	95	85	94	95	95	83	92
Compression depth score (%)	N/A	94	99	99	92	95	96	96
Depth (mm)	N/A	62	65	68	63	67	65	66
Average rate (compressions/min)	N/A	109	109	109	106	108	103	105
Compression in range (%)	N/A	92	89	94	88	93	89	93
Compression fraction (%)	N/A	100	100	99	100	99	99	100
Total score (**%**)	N/A	93	90	94	90	92	85	92

a N/A: not applicable.

No statistically significant difference in total CPR quality was observed immediately after zone 1 training (*P*=.83). However, significant differences emerged at 3 months (*P*<.001, large effect) and at 6 months (*P*<.001, large effect), both favoring the experimental group, suggesting better CPR quality retention over time. Full inferential statistics are presented in Table 5.

**Table 5.** Between-group comparisons of total cardiopulmonary resuscitation (CPR) quality scores at each assessment time point.

Time period	Control, n	Mean (SD) score control, %	Experimental, n	Mean (SD) score experimental, %	*U* ^a^	*z* ^b^	*P* Value^c^	*r* ^d^
Zone 1	19	90 (16.4)	28	94 (11.8)	256.5	−0.220	.83	−0.032
3 months	14	90 (12.1)	24	92 (10.9)	77.0	−3.350	<.001	−0.543
6 months	13	85 (17.8)	23	92 (10.9)	38.0	−3.881	<.001	−0.647

a *U*: Mann-Whitney *U* statistic.

b *z*: standardized test statistic.

c *P* values are 2-tailed.

d *r*: effect size (*r*=Z/√N).

## Discussion

### Summary of Main Findings

This pilot randomized educational trial examined whether the addition of a zone 0 self-directed preparatory phase using IV could enhance CPR competence acquisition and retention among nursing students while also assessing the feasibility and acceptability of the intervention as complementary pilot objectives. Compared with instructor-led training alone, adding this preparatory phase was feasible, well accepted, and free of adverse events or technical issues. The experimental group showed numerically higher CPR competence than the control group at all assessment time points, with the difference reaching statistical significance at 6 months. CPR quality scores were comparable between groups immediately after training but were significantly higher in the experimental group at 3 and 6 months, suggesting a meaningful advantage in skill retention over time.

### Interpretation and Comparison With Existing Literature

The integration of IV in zone 0 allows students to control their learning pace, review key steps as needed, and actively engage in their training process. These features are consistent with prior research on video-based procedural training, which has shown that such tools can improve the acquisition of complex clinical skills [33,34]. The integration of standardized instructional videos into the early stages of training appears to enhance comprehension and procedural memory, contributing to the superior performance observed in the present study.

These findings align with previous research supporting the effectiveness of the 4-step methodology developed by Peyton in teaching clinical skills. A meta-analysis by Nikendei et al [33], along with studies comparing video-based versions of the method developed by Peyton with other approaches [34,35], highlights its efficacy and versatility across diverse educational contexts. Seifert et al [34] reported that a video-based version of the 4-step method developed by Peyton was more effective than the traditional “see one, do one” approach in teaching complex surgical skills. In the specific context of CPR, a quasi-experimental study comparing the Kolb and Peyton educational models in CPR knowledge and performance among nurses found that the group using the methodology developed by Peyton showed significantly greater improvement in CPR performance [35].

The pattern of increasing between-group differences over time, reaching statistical significance at 6 months, is consistent with a preliminary signal of intervention effectiveness, particularly in relation to skill retention. The combination of the structured methodology developed by Peyton with self-directed learning in zone 0 appears to offer a more complete and effective learning experience for nursing students. This approach not only facilitates the acquisition of technical competencies but also promotes active and autonomous learning, which is essential in clinical education. Complementing this constructivist framework, Bandura [19] self-efficacy theory highlights the importance of perceived competence as a motivational driver in self-directed learning contexts. Zone 0 environments, which require learners to regulate their own practice without immediate instructor support, may be particularly influenced by students’ initial self-efficacy beliefs, which may partly explain the sustained retention observed in the experimental group.

To our knowledge, this is the first study to directly compare zone 0 combined with zone 1 with zone 1 alone in the acquisition and retention of CPR competencies. The results suggest that this methodology contributes to better skill acquisition and retention, as well as higher-quality performance of the resuscitation technique. This pilot study lays the foundation for future research following this methodology.

The present study assessed technical CPR competence as its primary educational outcome. However, simulation-based learning encompasses broader dimensions including student engagement, motivation, and perceptions of the learning environment, which may also be influenced by zone 0 preparatory phases. Future studies should consider incorporating validated multidimensional, simulation-based learning evaluation instruments, such as the CHEST (Comprehensive Healthcare Education Simulation Tool), recently validated in nursing education contexts [36], to provide a more comprehensive assessment of how preparatory self-directed learning phases affect the overall simulation experience beyond technical skill acquisition.

### Limitations

This pilot study has several limitations. The small sample size and single-center design limit the precision of estimates and the generalizability of the findings, although 95% CIs and effect sizes have been calculated to support interpretation. The follow-up period was limited to 6 months. Future studies should assess skill retention at intervals consistent with professional recertification requirements [37,38].

All training sessions and assessments were delivered by a single ERC/AHA-certified instructor, which ensured consistency but may have introduced instructor-related bias. Interrater reliability was not calculated, as only one evaluator performed all assessments; future studies should establish interrater agreement using Cohen κ or the intraclass correlation coefficient.

The inequality in total instructional time between groups (approximately 30 additional min for the experimental group) means it is not possible to fully disentangle the specific effect of IV learning from additional time-on-task. Future studies should equalize total contact time or include a time-matched active control condition.

A difference in age distribution was observed between groups, with the control group entirely within the 18 to 22 year range and 33% (11/33) of the experimental group participants aged 23 years or older. Age was collected using grouped response categories rather than exact continuous values, and cell counts in several categories were small; therefore, formal statistical comparison was not possible. Older students may have different self-directed learning dispositions that could have influenced outcomes independently of the intervention.

The study design did not include a zone 0–only group due to curricular constraints, which prevented isolation of the specific contribution of each training phase. Finally, the study assessed only technical CPR competence and did not include measures of student engagement, motivation, or perceived quality of the learning environment. Future studies should incorporate validated multidimensional, simulation-based learning evaluation instruments, such as the CHEST [36], to capture the full spectrum of educational outcomes.

### Conclusions and Broader Implications

This pilot randomized educational trial suggests that integrating a self-directed SimZone 0 preparatory phase using IV before instructor-led CPR training is feasible, acceptable, and associated with preliminary signals of higher CPR competence retention compared with instructor-led training alone, with between-group differences reaching statistical significance at 6-month follow-up. These findings should be interpreted with caution given the pilot nature of the study, the small sample size, the single-center design, and the inequality in total instructional time between groups.

The findings support the potential value of incorporating structured preparatory self-directed learning into CPR education for undergraduate nursing students. If confirmed in larger studies, this approach could represent a scalable and resource-efficient strategy for improving CPR skill acquisition and retention in health sciences education. Larger multicenter randomized trials with equal contact time between groups, larger and more diverse samples, and longer follow-up periods are required to confirm effectiveness, optimize instructional sequencing, and evaluate the generalizability of this approach across other procedural skills and educational contexts.

## Supplementary material

10.2196/95529 Checklist 1 CONSORT checklist.

## Abbreviations

AHA: American Heart Association
CHEST: Comprehensive Healthcare Education Simulation Tool
CONSORT: Consolidated Standards of Reporting Trials
CPR: cardiopulmonary resuscitation
ERC: European Resuscitation Council
IV: interactive video
OR: odds ratio
